# Potential Pathways and Genes Involved in Lac Synthesis and Secretion in *Kerria chinensis* (Hemiptera: Kerriidae) Based on Transcriptomic Analyses

**DOI:** 10.3390/insects10120430

**Published:** 2019-11-28

**Authors:** Weiwei Wang, Pengfei Liu, Qin Lu, Xiaofei Ling, Jinwen Zhang, Ming-Shun Chen, Hang Chen, Xiaoming Chen

**Affiliations:** 1 Research Institute of Resource Insects, Chinese Academy of Forestry, Kunming 650224, China; sk121ww@163.com (W.W.); liupengfei2010@aliyun.com (P.L.); luqin0319@126.com (Q.L.); lingxf309@163.com (X.L.); 13888748915@139.com (J.Z.); cafcxm@139.com (X.C.); 2 Department of Entomology, Kansas State University, Manhattan, KS 66506, USA; mchen@ksu.edu; 3 The Key Laboratory of Cultivating and Utilization of Resources Insects, State Forestry Administration, Kunming 650224, China

**Keywords:** *Kerria chinensis*, transcriptome, gene expression, lac synthesis

## Abstract

Lac is a type of natural resin secreted by lac insects and is widely used in the military and other industries because of its excellent adhesion and insulation properties. The main ingredients of lac are lactones and lactides, which are formed from hydroxy fatty acids and sesquiterpene esters. In this study, we measured lac secretion rates by the insect *Kerria chinensis* at different developmental stages and identified lac secretion-minimum and lac secretion-active stages of the insect. We then analyzed transcriptomes of lac secretion-minimum and lac secretion-active stages of the insect. Based on expression profiles of genes in different stages of the insect, we identified pathways and genes that are potentially involved in lac synthesis and secretion in *K. chinensis*. Our study lays a foundation for future studies to reveal the molecular mechanisms and pathways of lac synthesis and secretion in this beneficial insect.

## 1. Introduction

Lac is a mixture of natural resins produced in and secreted by insects from the genius *Kerria* (Hemiptera: Kerriidae) [1]. The use of lac by humans can be dated back to centuries ago. Initially, lac was mainly used as wood finishing and as an adhesive for ceramics. At present, lac is used in the production of music instruments, insulation of electronics, coating of pharmaceutical pills, painting, material conservation, and various applications in the military industry [1]. Due to its strong adhesiveness, good insulation, moisture resistance, smooth film texture, non-toxic properties, and tastelessness, lac is expected to be continuously widely used in various industries in the future [2,3].

The composition of lac derived from different insect species varies. Host trees and environmental conditions can also affect the chemical composition of lac secreted even from the same insect species. In general, lac is mainly composed of lac resins, shellac waxes, laccaic acids, and other organic chemicals [2]. Lac resins are mixtures of lactones and lactides, which are formed from hydroxyl fatty acids and polyterpene esters [2].

The biosynthetic pathway for polyterpene esters is the mevalonate pathway (MVA) in the cytoplasm [4,5]. In the mevalonate pathway, acetyl-CoA is converted into mevalonic acids by the action of hydroxymethylglutaryl-CoA synthase and hydroxymethylglutaryl-CoA reductase. Mevalonic acids are then converted to sesquiterpene acids by the action of terpene synthases (TPS). Sesquiterpene acids are then esterified to form sesquiterpene acid esters (Figure S1) [6]. Hydroxyl fatty acids are produced via the fatty acid synthesis pathway, which include steps catalyzed by fatty acid synthetases (FAS), elongases of very long chain fatty acid (ELO), and fatty acid desaturases (FAD) (Figure S1). Aleuritic acid (-9,10,16-erythro-aleuritic acid) is another important component of lac resins, and is synthesized from acetyl-CoA, again by fatty acid synthases [7].

**Figure 1:**
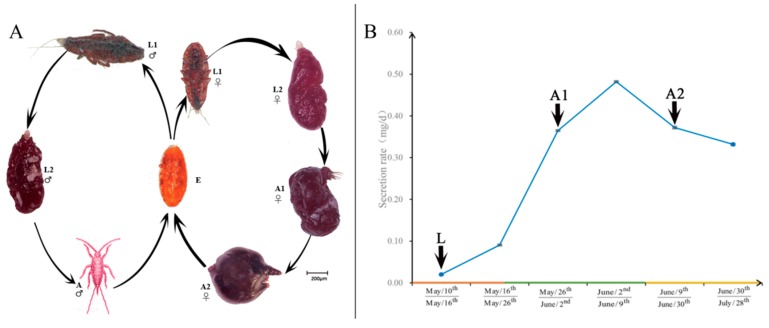
Life cycle and lac secretion stages. (**A**) Life cycles and sexual dimorphism of *Kerria chinensis*. The life cycle and morphology of males (Figure 1A, left part) and females (Figure 1A, right part) are different. Males have four life stages, including eggs (E), early instar larvae (L1), late instar larvae (L2), and adults (A). Females have five stages, including eggs, early instar larvae, late instar larvae, early stage adults (A1), and mid–late stage adults (A2). Morphologically, males and females are very different, especially at the adult stages. (**B**) Lac secretion of females at different stages. The Y-axis shows lac secretion rates and the X-axis shows the time when samples were collected. The arrows indicate the times and stages of samples collected for transcriptomic analyses.

The genius *Kerria* contains several species that are capable of secreting lac, including *Kerria chinensis*, *K. lacca*, *K. yunnanesis*, *K. sindica*, *K. ruralis*, *K. pusana*, and *K. nepalensis*. Different species are distributed in different regions, including Thailand, Myanmar, India, and China. One of the main species for lac secretion in South China is *K. chinensis* [2]. *K. chinensis* is an insect with distinct sexual dimorphism. Males and females are not only remarkably different in morphology and life cycle (Figure 1A), but also different in their ability to secrete lac. *K. chinensis* is born in two generations, including summer and winter generations, per year. The summer generation takes place from April to August, and the female first instar larvae is about 20 d, the second instar larvae is about 30 d, and the adult is about 90 d; the male instar larvae is about 20 d, the second instar larvae is about 20 d, the pupa is about 12 d, and the adult is about 8 d. The winter generation takes place from September to the following March and, the female first instar larvae stage is about 50 d, the second instar larva stage is about 70 d, the adult stage is about 90 d, the male first instar larvae stage is about 50 d, the second instar larvae stage is about 60 d, the pupal stage is about 20 d, and the adult stage is about 15 d [1]. In the past few decades, scientists have made progress on revealing the polymerization and resin composition of lac produced by *K. chinensis*. Several studies were also carried out to characterize biology, ecological adaptability, host plants, and genetic diversity of lac insect [8,9,10,11,12]. However, the molecular mechanisms and biochemical pathways for lac synthesis and secretion are not yet known. *K. chinensis* is also a genomically understudied insect species, and no genome sequence nor transcriptomic data are available.

In recent years, high-throughput sequencing technology has been widely used in gene mining and functional identification of various organisms. Insect transcriptome research has yielded fruitful results [13,14], such as *Ericerus pela* and *Schlechtendalia chinensis* [15,16,17]. However, the *K. chinensis* transcriptome was not reported, especially lac secretion genes. The objectives of this study are: (1) to generate transcriptomes with samples from different developmental stages of the insect for gene identification; and (2) to initially identify genes and pathways that are potentially involved in lac synthesis and secretion based on the transcriptomic data.

## 2. Materials and Methods

### 2.1. Insects

*K. chinensis* was reared on *Schleichera oleosa* in the experimental station of Yuanjiang, Research Institute of Resource Insects, Yunnan province, China (102°00’46” E, 23°36´11” N). The female lac insects from larval stage (L), early adult stage (A1), and mid–late adult stage (A2) were collected and used for RNA extraction, library construction, and sequencing. Similar samples were also prepared for quantitative PCR analysis, and for individual assays to determine lac secretion rates.

### 2.2. Measurement of Lac Secretion Rates

Branches of host plants colonized densely with *K. chinensis* were cut and harvested as sticklac. The harvested sticklac was soaked in 95% alcohol until lac was completely dissolved into the alcohol solution, which was then filtered to separate insects and lac. Individual insects were counted and weighed. The weight of crude lac was derived by subtracting the weight of insect bodies from the sticklac weight. The average crude lac secreted per insect was obtained through dividing total crude lac by the number of insects. The rate of individual secretion was obtained through dividing the difference between crude lac secretion on the initial collection date and crude lac secretion on the final collection date by the number of days. Three different stage female samples were measured, and three independent biological replicates were conducted for each measurement.

The average amount of lac secreted by individual insects was calculated by Formula (1), whereas the individual secretion rate was calculated by Formula (2).

(1) $$M=\frac{m_{1}-m_{2}}{N}$$

In Formula (1), *M* represents the amount of individual secretion of the lac insects; *m*_1_ represents the total weight of lac and insects in a lac block (containing lac and insects); m_2_ represents the weight of insects in the lac block; and *N* represents the number of insects in the lac block.

(2) $$v=\frac{M_{\left(n+1\right)}-M_{n}}{t_{\left(n+1\right)-}t_{n}}$$

In the Formula (2), *v* represents the individual secretion rate; *M_n_* represents the amount of individual secretion in the nth time; *M*_(*n*+1)_ represents the amount of individual secretion in the (*n*+ 1)th time. *T_n_* represents the nth acquisition time; and *T*_(*n*+1)_ represents the (*n*+1)th acquisition time.

### 2.3. RNA Extraction and Sequencing

Total RNA from L, A1, and A2 stages was extracted using Trizol Reagent (Sangon Biotech, Shanghai, China) according to the method provided by the manufacturer. RNA quality was monitored on 1% agarose gels. RNA quantification and integrity were checked using a NanoPhotometer^®^ spectrophotometer (IMPLEN, Westlake, CA, USA), and a Qubit^®^ RNA Assay Kit (Sangon Biotech, Shanghai, China) in a Qubit^®^ 2.0 Flurometer (Thermo Fisher Scientific, Waltham, MA, USA), and an RNA Nano 6000 Assay Kit (Sangon Biotech, Shanghai, China) on an Agilent Bioanalyzer 2100 system (Agilent Technologies, Santa Clara, CA, USA), respectively. A total amount of 3 μg RNA per sample was used for mRNA enrichment using magnetic beads with Oligo (dT). mRNA species were fragmented into short pieces with average size 150~200 bp. The fragmented mRNA pieces were used as templates for reverse-transcription with random hexamer primers and M-MuLV Reverse Transcriptase (RNaseH)- to synthesize the first strand cDNA. Second strand cDNA was synthesized subsequently using DNA Polymerase I (Sangon Biotech, Shanghai, China) and RNase H (Sangon Biotech, Shanghai, China). Remaining overhangs were converted into blunt ends via exonuclease/polymerase of polymerase I (Sangon Biotech, Shanghai, China). After adenylation of 3′ends of the DNA fragments, AMPure XP beads were used for fragment size selection. cDNA libraries were obtained by PCR amplification, and the PCR products were purified by AMPure XP beads. Quality of the libraries was measured by Qubit2.0, Agilent2100, and q-PCR. After cluster generation, the libraries were sequenced on an Illumina Hiseq^TM^2500 platform, and paired-end reads were generated.

### 2.4. Data Analysis

After sequencing, raw reads were subjected to credibility analysis. Sequences with a linker, sequences with N greater than 10%, and low-quality sequences (the number of bases with a mass value of Qphrd ≤ 5 more than 50% of the entire reads) were removed and discarded, and the remaining reads were referred as Cleans Reads. Q20, Q30, GC-content, and sequence duplication levels of the clean reads were calculated. All the downstream analyses were based on clean reads data. Transcriptome assembly was accomplished based on the de novo splicing of valid sequences [18]. The transcript sequence information was stored in FASTA format as a reference sequence for subsequent analysis using Trinity.

### 2.5. De Novo Assembly and Gene Annotation

Assembled unigenes were annotated by blasting against the NCBI non-redundant protein (Nr) database, NCBI nucleotide database (Nt), Swiss-Prot protein database (Swiss-Prot), Protein families database (PFAM), Kyoto Encyclopedia of Genes and Genomes (KEGG) database, Clusters of Orthologous Groups (COGs) database, and Gene Ontology (GO) database [19,20]. The e-value thresholds for Nr, Nt, and Swiss-Prot databases were 1e-5 and for KOG/COG database 1e-3. GO functional annotation was done using blast2go (b2g4pipe_v2.5). Annotation against the KEGG database was carried out using the KEGG Automatic Annotation Server (KAAS) with default parameters [21].

### 2.6. Quantitative PCR (qPCR) Analysis

To validate the results of RNA-seq analysis, several genes were selected from *K. chinensis* transcriptome data to perform qPCR experiments. The list of gene-specific primers is shown in Table S1. Each reaction was carried out with 1 μL of a 1/20 (v/v) dilution of the first cDNA strand, 0.5 mM of each primer in a total volume of 25 μL. Amplification was performed with iQ SYBR Green Supermix (Sangon Biotech, Shanghai, China) on an iCycler real time detection system (Bio-Rad, Hercules, CA, USA) as the following thermal circles: 95 °C for 30 s followed by 40 cycles of 95 °C for 5 s and 60 °C for 30 s. The unigene expression levels were calculated with the 2^−ΔΔCT^ method [22]. Actin was selected as a reference for normalization of template concentration. Three independent biological replicates were carried out for each treatment.

### 2.7. Analysis of Differential Expression Gene (DEG)

Read counts were used to estimate the levels of gene expression. In order to reduce false positives, trimmed mean of M values (TMM) was used to standardize data processing. Differentially expressed genes were identified by DEGseq with q value < 0.005 and log2 (fold change) > 1 [23,24].

### 2.8. Data Availability 

We have deposited our data in the Sequence Read Archive (SRA) (http://www.ncbi.nlm.nih.gov/sra/); the accession for our submission is PRJNA489372.

## 3. Results

### 3.1. The Characteristics and Dynamic Regularity of K. chinensis

Male and female larvae can only secrete a small amount of lac. The female is able to secret vast amounts of lac in the adult stage, whereas the male cannot secret lac any more both in the pupal and adult stages [1]. The lac secretion of *K. chinensis* in the whole summer generation was about 24.9 mg/female (Figure S2). Through the determination of individual gum secretion rate in each stage, the results showed that the individual gum secretion rate was slower in the larval stage and increased gradually in the adult stage. In the early adult stage (A1), the individual fastest secretion rate was 4.82 × 10^−1^ mg/d (Figure 1B), and the secretion decreased gradually in the mid–late adult stage (A2).

### 3.2. Overview of Transcriptomic Analyses

Based on the characteristics and dynamic regularity of *K. chinensis* observations, a strategy of transcriptomic analyses was designed to identify candidate genes and pathways that are potentially involved in lac synthesis and secretion (Figure S3). The strategy was conducted to identify genes that are differentially expressed between lac secretion-minimum stage (L) versus lac secretion-active stages (A1 and A2). Those genes that are commonly up- or down-regulated in A1 and A2 in comparison with L are potentially involved in lac synthesis and secretion. Those genes that are differentially regulated between A1 and A2 in comparison with L are likely involved in other developmental regulations.

Illumina sequencing of cDNA libraries generated a total of 24,897,365 raw reads covering a total of 126,059,825 nucleotides. After removing adapter sequences, ambiguous nucleotides and low-quality sequences, 24,455,197 high quality reads were retained. The mean length of reads was 486 nt. The high quality reads were assembled into 183,899 unigenes, which had average size of 532 nt and N50 670 nt. Detailed statistics of spliced transcripts and assembled unigenes are shown in Table S2 and Figure S4. These statistics were comparable with those of high standard transcriptomes from other well known insect species.

To annotate the unigenes, a blastx search was carried out against the NR database with a cutoff E-value smaller than 10^−5^ (Table 1). A total of 54,037 unigenes (29.38% of all unigenes) returned above cutoff alignments when searched against the NR nucleotide database. Specifically, 71.6% of matched sequence alignments had E-values ranging between 1.0E^−5^ and 1.0E^−60^, and the remaining alignments were with E-values less than 1.0E^−60^ (Figure S5). The similarity of 7.2% sequence alignments ranged from 96% to 100%, 24.0% ranged from 80% to 95%, 46.9% ranged from 60% to 80%, 21.8% ranged from 40% to 60%, and only 0.1% ranged from 18% to 40% (Figure S5). The species distribution of the first hits was 10% with *Acyrthosiphon pisum*, 9.3% with *Zootermopsis nevadensis*, 7.2% with *Nasonia vitripennis*, 4.2% with *Diaphorina citri*, and 3.7% with *Bombyx mori* (Figure S5). In addition, the unigenes were similarly analyzed against other databases, and the results are summarized in Table 1 as well.

Of the 183,899 unigenes, 50,406 were assigned to specific GO terms and classified into 19 subcategories of ‘biological processes’, 15 subcategories of ‘cellular components’, and 12 subcategories of ‘molecular functions’ (Figure S6). The subcategories of ‘cellular process’, ‘cell’, and ‘binding’ were dominant in each of the three major categories, respectively. Analysis of the unigenes against the KEGG database, a network diagram of cell metabolic pathways, assigned 30,464 unigenes specific pathways (Figure S7). Among them, 15,460 unigenes were annotated to ‘metabolism’ (40.07% of the total), 3839 to ‘environmental information processing’ (9.95%), 7929 to ‘genetic information processing’ (20.55%), 7162 to ‘organismal systems’ (18.56%), and 4011 to ‘cellular processes’ (10.47%).

Analysis of the unigenes against the KO database classified 30,464 unigenes (16.56% of all unigenes) to 32 KEGG pathways after removing unigenes associated with the human diseases. Among the classified unigenes, 272 were annotated to ‘metabolism of terpenoids and polyketides pathway’, include 142 unigenes involved in ‘terpenoid backbone biosynthesis (PATHWAY: ko00900)’, 10 unigenes involved in ‘sesquiterpenoid and triterpenoid biosynthesis (PATHWAY: ko00909)’, one unigene involved in ‘monoterpenoid biosynthesis (PATHWAY: ko00902)’, one unigene involved in ‘diterpenoid biosynthesis (PATHWAY: ko00904)’ (Figure S8). In addition, 2197 unigenes were annotated to ‘lipid metabolism’, including 199 unigenes involved in ‘unsaturated fatty acids biosynthesis (PATHWAY: ko01040)’, 218 unigenes in ‘fatty acid biosynthesis (PATHWAY: ko00061)’, 130 unigenes in ‘fatty acid elongation (PATHWAY: ko00062)’, and 311 unigenes in ‘fatty acid degradation (PATHWAY: ko00071)’. The unigenes were also classified into Clusters of Orthologous Groups (COG) (Figure S9). A total of 36,724 (19.96%) unigenes were subdivided into 26 COG classifications. Among them, the cluster of ‘general function prediction’ (5364, 14.61%) represented the largest group, followed by ‘posttranslational modification, protein turnover, chaperones’ (4399, 11.98%), ‘translation, ribosomal structure, and biogenesis’ (3961, 10.79%), and other categories (less than 3000).

### 3.3. Genes Expressed Differentially between Insects at Different Stages

**Figure 2:**
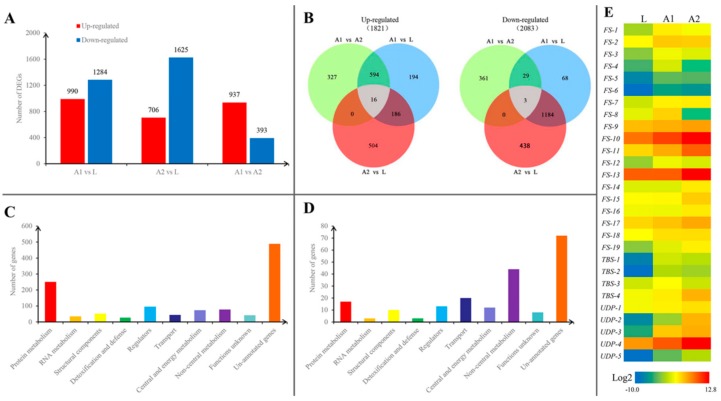
Genes expressed differentially at different developmental stages and candidate genes putatively involved in lac synthesis. (**A**) The numbers of differentially expressed genes (DEGs) between lac secretion-minimum and lac secretion-active stages. L is the lac secretion-minimum stage whereas A1 and A2 are lac secretion-active stages. (**B**) Venn diagram showing the number of commonly up- and down-regulated genes among the three different stages of the insect. (**C**) Composition of commonly up-regulated. (**D**) Composition of commonly down-regulated. (**E**) The heatmap of expression profiles of 28 DEGs in different developmental stages of lac insect.

There were 2774 genes expressed differentially between A1 and A2, with 990 genes up-regulated and 1284 down-regulated. There were 2331 genes expressed differentially between A2 and L, with 706 genes up-regulated and 1625 down-regulated. There were 1330 genes expressed differentially between A1 vs. L, with 937 genes up-regulated and 393 down-regulated (Figure 2A). Combinational analyses revealed that 202 genes were commonly up-regulated and 1187 genes commonly down-regulated in the two lac secretion-active adult stages in comparison with the lac secretion-minimum larval stage (Figure 2B). The remaining genes were differentially regulated among the three stages. Since the commonly up- and down-regulated genes are most likely involved in lac synthesis and secretion, the composition of these genes were analyzed in detail in the following sections.

### 3.4. Reduced Protein Synthesis in Lac Secretion-Active Stages

As shown in Figure 2C,D, the largest proportions of genes that were both commonly up- and down-regulated are un-annotated. These unique genes represent opportunities to identify novel genes with new functions. The roles of these un-annotated genes in lac synthesis and secretion remain to be studied.

The second largest category of genes that were commonly down-regulated was ‘protein metabolism’, which included proteins involved in protein synthesis, folding, modification, and degradation (Table 2, Table S3). There were 21% of commonly down-regulated genes belong to the ‘protein metabolism’ category. On the other hand, there were only 8% of commonly up-regulated genes belong to this category. Most of the commonly down-regulated genes encode various ribosomal proteins, followed by chaperones, and proteases or protein degradation-related, indicating that protein synthesis was at much lower levels in lac secretion-active stages of the insect. In comparison, most of the commonly up-regulated ‘protein metabolism’ genes encode proteins involved in folding and degradation (Table S4). The combination of down-regulation of genes in protein synthesis and up-regulation in protein degradation indicated an overall reduction in protein synthesis in lac secretion-active stages. This could be a way for the insect to save energy and nutrients for the production and secretion of lac.

### 3.5. Reduced and Unbalanced Central Metabolic Pathways

Overall, metabolism via central metabolic pathways was reduced in insects at lac secretion-active stages based on the proportion of genes that are commonly up- or down-regulated (Table 2, Figure S10). There were 70 down-regulated genes involved in central metabolic pathways and respiratory chain with average of 5.59 log2 fold down-regulation. In comparison, there were only 22 genes up-regulated with only 1.25 log2 fold increase. However, not every step in the three central metabolic pathways was affected evenly. All (37) identified genes encoding components in the respiratory chains were down-regulated with similar fold changes, indicating that overall ATP production for energy use was lowered. However, there were genes for some enzymatic steps, which were actually up-regulated (Table 2, Figure S10). This uneven regulation of central metabolic pathways suggested that these pathways were regulated for accumulation of certain intermediates for biosynthesis. Presumable accumulated intermediates include glucose-6-phosphate, ribulose-5-phophate, glyceraldehide-3-phosphate, sedoheptulose-7-phosphate, glucose-6-phosphate, fructose-1,6-bisphosphate, citrate, succinyl-CoA, and malate. These are activated sugars and other intermediates that could be used for lac synthesis directly or indirectly.

### 3.6. Shifted Metabolism of Fatty Acids, Lipids, and Terpenes

In contrast to the overall reduction in protein metabolism and unevenly affected central metabolic pathways, the metabolic pathways of fatty acids, lipids, and terpenes were shifted in the lac secretion-active stages in comparison with the lac secretion-minimum stage (Table 2). There were 24 genes commonly up-regulated and about an equal number (23) of genes down-regulated, even though the magnitude of down-regulation was much bigger than that of up-regulation. The up- and down-regulation of genes within the same functional categories indicated a shift in formation of different products of the same categories at different stages. We hypothesize that the genes up-regulated in the lac secretion-active stages are involved in lac production and secretion.

### 3.7. Candidate Genes Potentially Involved in Lac Synthesis and Secretion

**Figure 3:**
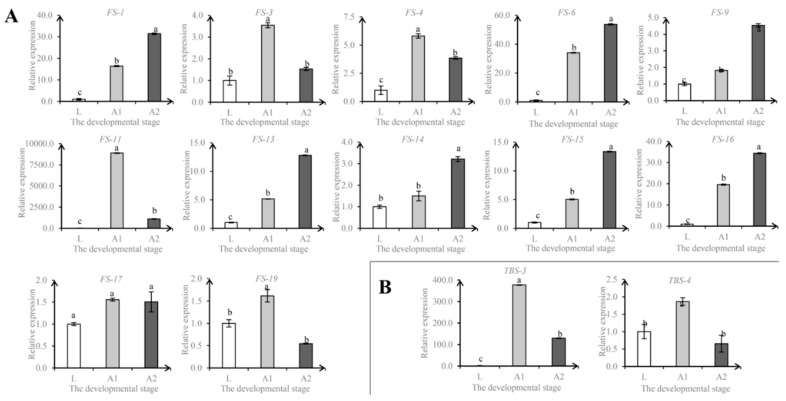
qPCR analyses of 14 DEGs in the two shellac secretion-active adult stages in comparison with the shellac secretion-minimum late larval stage. (**A**) The genes related to fatty acid synthesis. (**B**) The putative genes involved in terpenoid biosynthesis. Horizontal axis indicates sample collection stage. Small letters mean that the analysis of the significantly of DEGs at L, A1, and A2 stages (*p* < 0.05).

Based on the correlation between gene expression levels and lac accumulation rates as well as the putative functions of genes, candidate genes potentially involved in lac synthesis and secretion were identified, including genes involved in fatty acid synthesis: *FS-1* (acetyl-CoA carboxylase, fatty acid synthesis), *FS-8* (acyl-CoA reductase, fatty alcohol synthesis), *FS-1* (acyl-CoA synthetase, fatty acid biosynthesis), *FS-2* (acyl-CoA synthetase), *FS-7* (acyl-CoA synthetase), *FS-5* (fatty acid synthase), *FS-11* (fatty acid synthase), *FS-12* (fatty acid synthase), *FS-6* (fatty acid synthase), *FS-10* (ATP-citrate lyase, fatty acid biosynthesis), *FS-3* (fatty acid desaturase, fatty acid desaturation), *FS-4* (fatty acid desaturase), *FS-14* (fatty acid desaturase), *FS-13* (fatty acid desaturase), *FS-18* (fatty acyl-CoA elongase), *FS-17* (medium-chain acyl-CoA dehydrogenase, fatty acid oxidation), *FS-9* (17-beta hydroxysteroid dehydrogenase), *FS-16* (trans-2,3-enoyl-CoA reductase), and *FS-15* (enoyl-CoA hydratase); genes involved in terpenoid biosynthesis: *TBS-4* (hydroxymethylglutaryl-CoA synthase), *TBS-2* (polyprenyl synthetase), *TBS-1* (polyprenyl synthetase), and *TBS-3* (decaprenyl-diphosphate synthase); and genes involved in UDP-driving glycosylation: *UDP-1* (oligosaccharyltransferase, N-linked glycosylation pathway), *UDP-2* (UDP-glucuronosyl/-glucosyl transferase, catalyzes the addition of the glycosyl group from a UTP-sugar to a small hydrophobic molecule), *UDP-3* (UDP-glucuronosyl/-glucosyl transferase), *UDP-4* (UDP-glucuronosyl/-glucosyl transferase), and *UDP-5* (UDP-glucuronosyl/-glucosyl transferase). As shown in Figure 2E, Table S5, the expression of these genes was well correlated with the secretion of lac. The genes putatively involved in fatty acid synthesis may produce hydroxyl fatty acids contained in lac. The genes putatively involved in terpenoid synthesis may participate in production of sesquiterpene acids, which are major components of lac [1]. The involvement of UDP-dependent glycosylation in lac synthesis remains very speculative. The reason we think it might be involved directly or indirectly is the apparent accumulation of activated sugars based on unbalanced central metabolic pathways. The same types of genes are specifically up-regulated as well in wax secretion insects [15]. Among these differentially expressed genes, 14, namely *FS-1, FS-3, FS-4, FS-6, FS-9, FS-11, FS-13, FS-14, FS-15, FS-16, FS-17, FS-19, TBS-3*, and *TBS-4*, were confirmed by qPCR (Figure 3).

## 4. Discussion

A total of 205,581 transcripts were obtained in this study, among which 183,899 unigenes were successfully annotated by seven databases including Nt, Nr, Swiss-Prot, COG, GO, KOG, and KEGG. The most sequences were annotated in *A. pisum* by homologous sequence alignment in the Nr database; they may have a relatively close genetic relationship and the *A. pisum* has abundant genomic information [25]. There are 50,406, 36,724, and 30,464 unigenes annotated to GO, COG, and KEGG databases, respectively, which provide rich data resources for deeper studies on the mechanism of lac secretion of lac insects in the future.

The secretion rate of lac was different between different individuals. The secretion rate of *Kerria lacca* gradually decreased in the adult stage; the highest increase of secretion rate of each female was 2.54 × 10^−4^ mg/d in the early adult, which gradually decreased at middle and late stages [26]. At larval and late adult stages of *Kerria yunnanensis*, the individual secretion rate was relatively slow. In the middle adult stage, the maximum secretion rate was 1.46 × 10^−1^ mg/d, which gradually decreased at late stages [27]. Through the study on the secretion rate of *K. chinensis*, it was found that the mid–late stage had a maximum secretion rate of 4.82 × 10^−1^ mg/d, and the secretion rate gradually decreased at later stages. Therefore, the study proved that the expression of genes associated with secretion of lac in the adult stage was significantly different (especially higher than other stages), which will provide a foundation for screening the follow-up genes associated with lac biosynthesis.

Based on our results, we propose a hypothesis for lac synthesis in *K. chinensis* (Figure S11). The model consists of three major physiological changes that provide the basis for lac synthesis, including savings of nutrients and energy, production of intermediates and NADPH necessary for lac biosynthesis, and a shift in metabolism of fatty acids, lipids, and terpenes that results in reduction of fatty acids, lipids, and terpenes for other physiological uses but increase of these compounds for lac synthesis. To save nutrients and energy, the overall metabolism is lowered in lac secretion-active stages, particularly in protein synthesis and structural components. The central metabolic pathways, including pentose phosphate pathway, glycolysis, and TCA cycle, were unequally affected, leading to accumulation of activated simple sugars and other intermediates for lac synthesis. The shift in metabolism of fatty acids, lipids, and terpenes results in reduced production of these compounds in other part of tissues that are used for normal growth and development, but elevated production in the lac secretion glands for the production of lac. Our model hypothesis provides directions for further experimental tests in the future regarding lac synthesis in the insect *K. chinensis*.

## 5. Conclusions

In this paper, the amount of individual secretion and rate of *Kerria chinensis* as the object of study of lac secretion in different periods were measured to reveal the lac secretion characteristics and dynamics. By analyzing the transcriptomes of different stages of *K. chinensis*, we speculated the correlation of gene functions through the link of gene expression level and lac secretion rate; the results showed that 28 candidate genes that may be involved in lac synthesis were preliminarily screened; meanwhile, the results provided a scientific basis for the verification of molecular mechanisms related to lac secretion.

## Figures and Tables

**Table 1:** Annotation result statistics between unigenes and databases.

Database	Numbers of Unigene	Percent (%)
Nr	54,037	29.38
Nt	18,328	9.96
Swiss-Prot	49,924	27.14
PFAM	49,642	26.99
KEGG	30,464	16.56
COG	36,724	19.96
GO	50,406	27.4
Annotated in all databases	8950	4.86
Total unigenes	183,899	100

**Table 2:** Commonly up-regulated and down-regulated genes in the two lac secretion-active adult stages in comparison with the lac secretion-minimum larval stage.

Metabolic Pathway	Up		Down	
	Number	Average Log2 Fold	Number	Average Log2 Fold
**Protein metabolism: Synthesis, modification, and degradation**				
Ribosomal proteins	1	1.19	113	−5.66
Initiation and elongation factors	1	7.77	27	−5.52
Chaperones and protein folding	6	2.41	34	−5.90
Proteases and protein degradation related	9	1.66	75	−5.47
Others	0	-	1	−5.52
Total	17	3.26	250	−5.61
**Central metabolic pathways and energy metabolism**				
Glycolysis	2	1.03	7	−5.92
Citrate acid cycle (TCA cycle)	4	1.38	14	−5.12
Pentose phosphate pathways	3	1.55	3	−6.13
Respiratory chain and energy metabolism related	2	1.02	37	−5.81
Others	0	-	9	−4.99
Total	22	1.25	70	−5.59
**Non-central metabolic pathways**				
Metabolism of fatty acids, lipids, terpenes	24	1.96	23	−4.68
Metabolism of nitrogen compounds	9	2.01	22	−5.31
UDP-glucuronosyl and UDP-glucosyl transferase	4	1.72	0	
Other metabolism	6	2.03	32	−4.92
Total	43	1.93	77	−4.97

## Supplementary Materials

The following are available online at https://www.mdpi.com/2075-4450/10/12/430/s1. Figure S1: The pathways of terpenoid and fatty acid biosynthesis. (**A**) The procession of terpenoid biosynthesis. ATOT (acetoacetyl-CoA thiolase); HMGS (hydroxymethylglutaryl-CoA synthase); HMGR (hydrox-methylglutaryl-CoA reductase); MK (mevalonate kinase); PMK (phosphomevalonate kinase); MPD (diphos phomevalonatedecarboxylase); IDI (isopentenyl diphosphate isomerase); FPPS (farnesyl diphosphate synthase); TPS (terpene synthase). (**B**) The procession of Fatty acid biosynthesis, ACC (acetyl-coenzyme A carboxylase); FAS (fatty acid synthase); ELO (elongase of very long chain fatty acid); FAD (fatty acid desaturase), Figure S2: Lac secretion of females at different stages, Figure S3: The strategy to identify shellac-secretion related genes, Figure S4: Length distribution of unigenes of *K. chinensis*, Figure S5: Data of Nr classification. (**A**) The E-value distribution of the result of Nr annotation. (**B**) The similarity distribution of the result of Nr annotation. (**C**) The species distribution of the result of Nr annotation, Figure S6: GO classification analysis of unigenes in unigene. GO functions is showed in Y-axis. The X-axis shows the number of genes that have the GO function, Figure S7: KEGG classification analysis of unigenes in all-unigene. KEGG functions is showed in Y-axis. The X-axis shows the percentage of genes that have the KEGG function, Figure S8: Metabolism of terpenoids, polyketides, and lipid metabolism pathway. (**A**) Metabolism of terpenoids and polyketides pathway. (**B**) Lipid metabolism pathway. Metabolism pathway is shown in Y-axis. The X-axis shows the number of genes, Figure S9: COG function classification of unigenes. The horizontal coordinates are function classes of COG and the vertical coordinates are numbers of unigenes in one class. The notation on the right is the full name of the functions in X-axis, Figure S10: Central metabolic pathways. (**A**) Pentose phosphate pathways. (**B**) Glycolysis. (**C**) Citruate acid cycle. Blue numbers: the number of genes related to the pathways of pentose phosphate pathways, Glycolysis and citruate acid cycle enzymes in transcriptome data, Figure S11: The lac synthesis in *K. chinensis*; Table S1: The quantitative PCR primers of 14 candidate genes in *K. chinensis*, Table S2: Assembly quality of *K. chinensis* transcriptome, Table S3: The commonly down-regulated genes metabolic pathways, Table S4: The commonly up-regulated genes metabolic pathways, Table S5: FPKM of 28 putative genes related to fatty acid synthesis, terpenoid biosynthesis, and UDP-driving glycosylation.
