# Supplementary material for: Potential Pathways and Genes Involved in Lac Synthesis and Secretion in Kerria chinensis (Hemiptera: Kerriidae) Based on Transcriptomic Analyses
Source: Insects. 2019 Nov 28;10(12):430. doi: 10.3390/insects10120430 (PMC6956188; doi:10.3390/insects10120430)
Supplement: Supplementary file 1 [file insects-10-00430-s001.pdf]

Supplementary Information

Potential Pathways and Genes Involved in lac Synthesis and Secretion in *Kerria chinensis* (Hemiptera: Kerriidae) Based on Transcriptomic Analyses

Weiwei Wang[1], Pengfei Liu[1], Qin Lu[1], Xiaofei Ling[1], Jinwen Zhang[1], Ming-Shun Chen[2], Hang Chen[1,3]*

Xiaoming Chen[1,3]

[1] Research Institute of Resource Insects, Chinese Academy of Forestry, Kunming ,650224, China; sk121ww@163.com (W.W.); liupengfei2010@aliyun.com (P.L.); luqin0319@126.com (Q.L.); lingxf309@163.com (X.L.); 13888748915@139.com (J.Z.); cafcxm@139.com (X.C.)

[2] Department of Entomology, Kansas State University, Manhattan, KS 66506, USA; mchen@ksu.edu

[3] The Key Laboratory of Cultivating and Utilization of Resources Insects, State Forestry Administration, Kunming, 650224, China

* Corresponding author: stuchen6481@gmail.com.

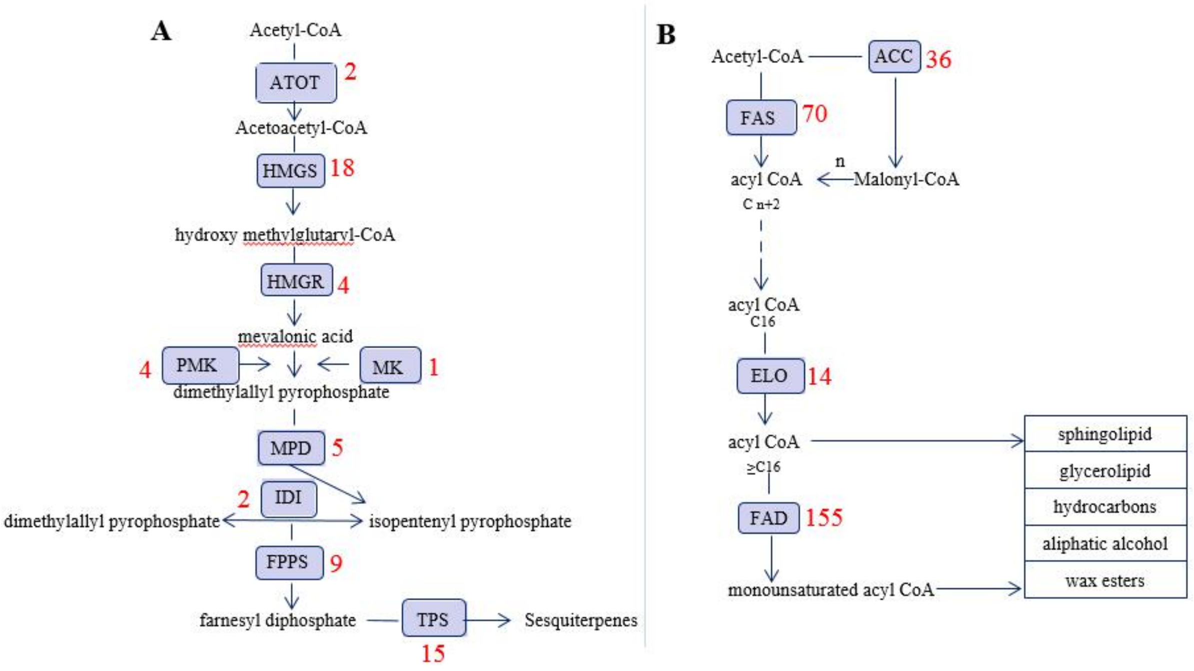

Figure S1.The pathways of Terpenoid and Fatty acid biosynthesis. (A) The procession of Terpenoid biosynthesis. ATOT (acetoacetyl-CoA thiolase); HMGS (hydroxymethylglutaryl-CoA synthase); HMGR (hydroxymethylglutaryl-CoA reductase); MK (mevalonate kinase); PMK (phosph-omevalonate kinase); MPD (diphosphomevalonate decarboxylase); IDI (isopentenyl diphosphate isomerase); FPPS (farnesyl diphosphate synthase); TPS(terpene synthase). (**B**) **The procession of Fatty acid biosynthesis,** ACC (acetyl-coenzyme A carboxylase); FAS (fatty acid synthase); ELO (long chain fatty acid elongase); FAD (fatty acid desaturase). Red numbers: the number of genes related to the pathways of Terpenoid and Fatty acid biosynthesis enzymes in transcriptome data.

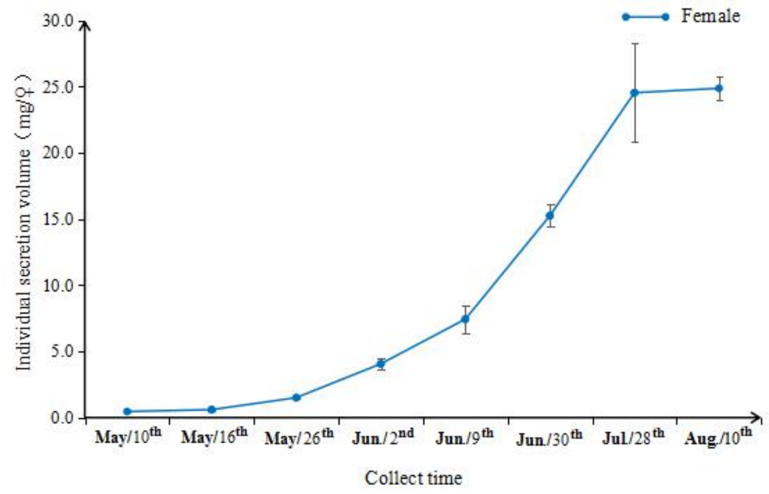

Figure S2. Lac secretion of females at different stages.

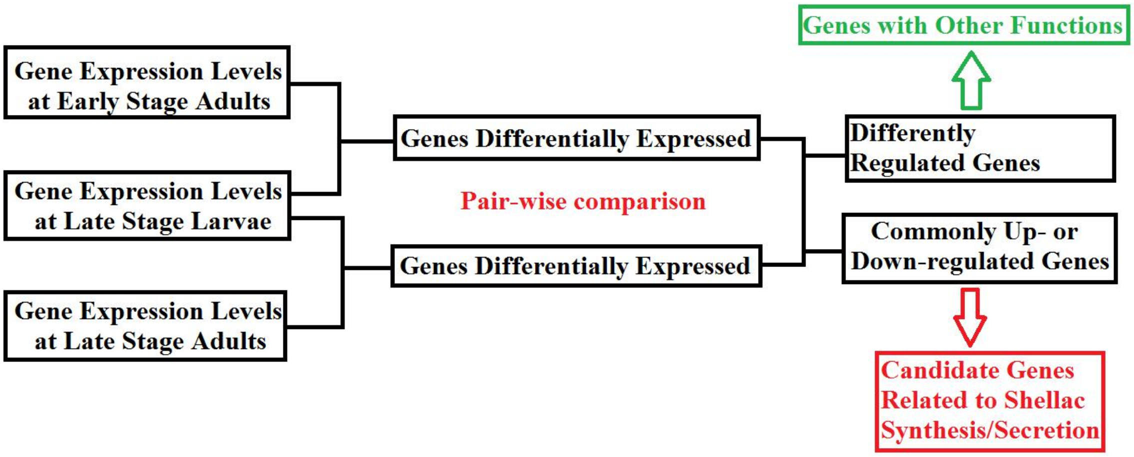

Figure S3. The strategy to identify shellac-secretion related genes.

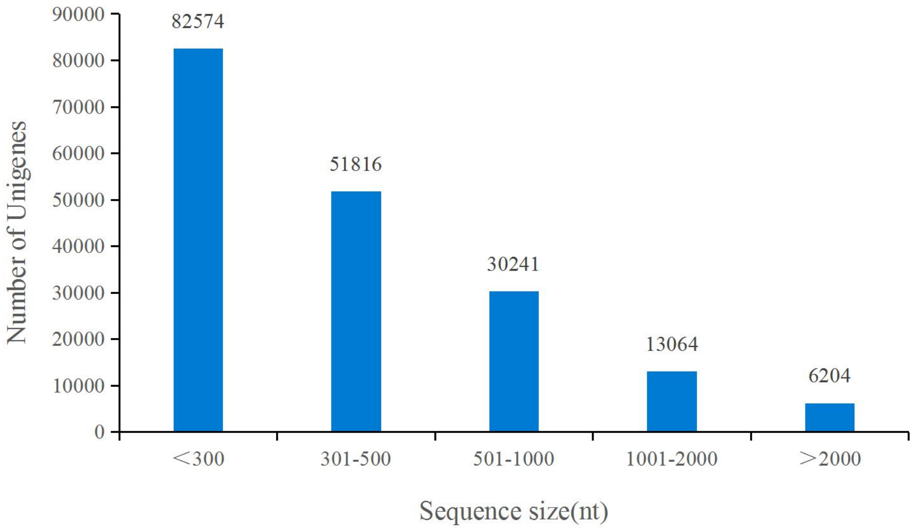

Figure S4. Length distribution of Unigenes of *K. chinensis*

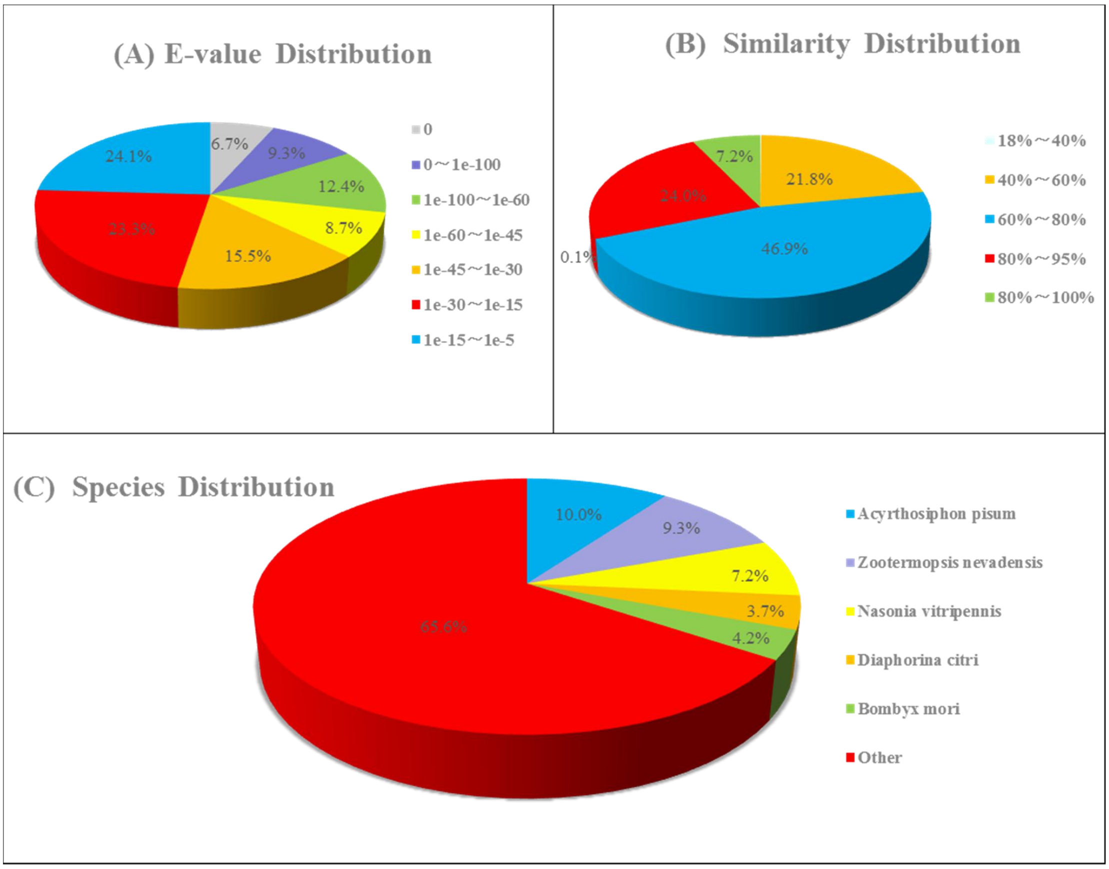

Figure S5. Data of Nr classification. (A) The E-value distribution of the result of Nr annotation. **(B)** The similarity distribution of the result of Nr annotation. **(C)** The species distribution of the result of Nr annotation.

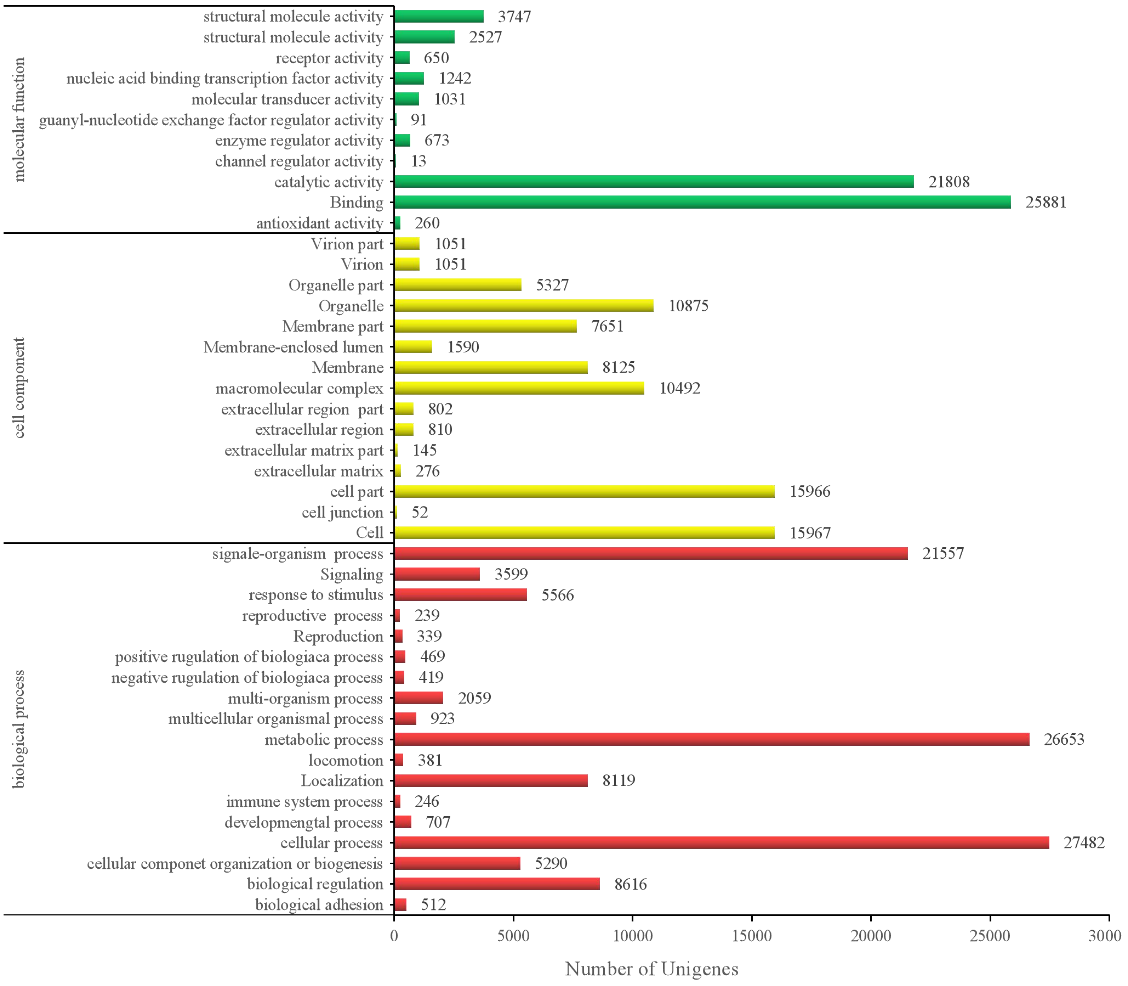

Figure S6. GO classification analysis of Unigenes in Unigene. GO functions is showed in Y-axis. The X-axis shows the number of genes which have the GO function.

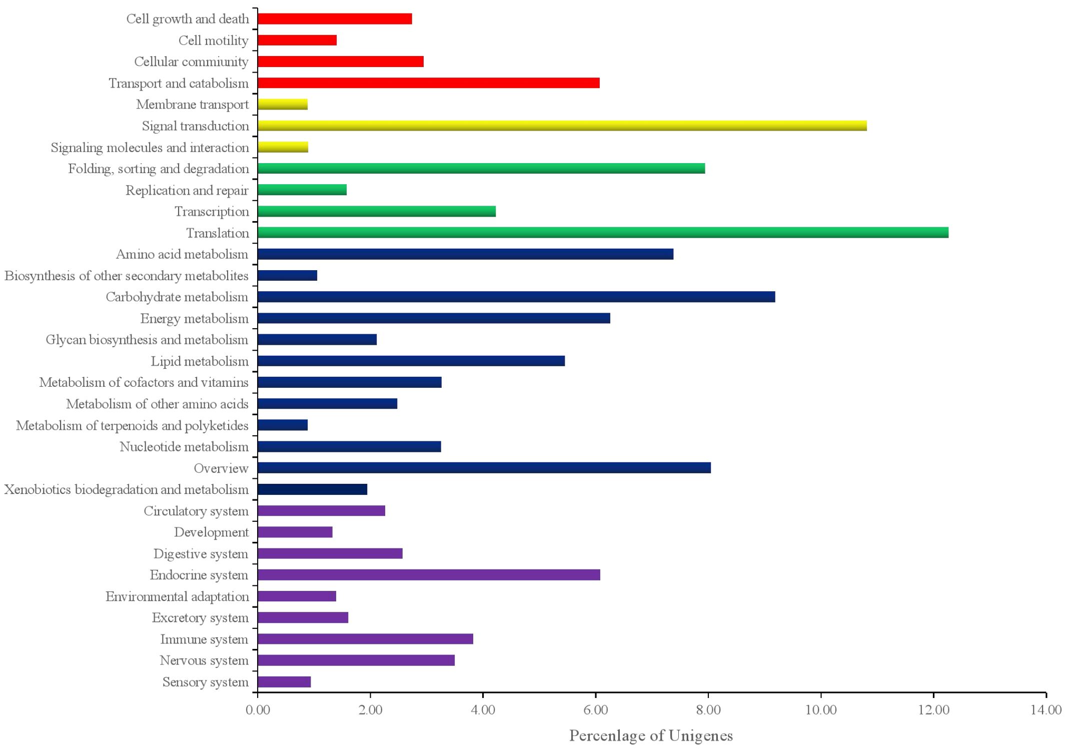

Figure S7. KEGG classification analysis of Unigenes in All-Unigene. KEGG functions is showed in Y-axis. The X-axis shows the percenlage of genes which have the KEGG function.

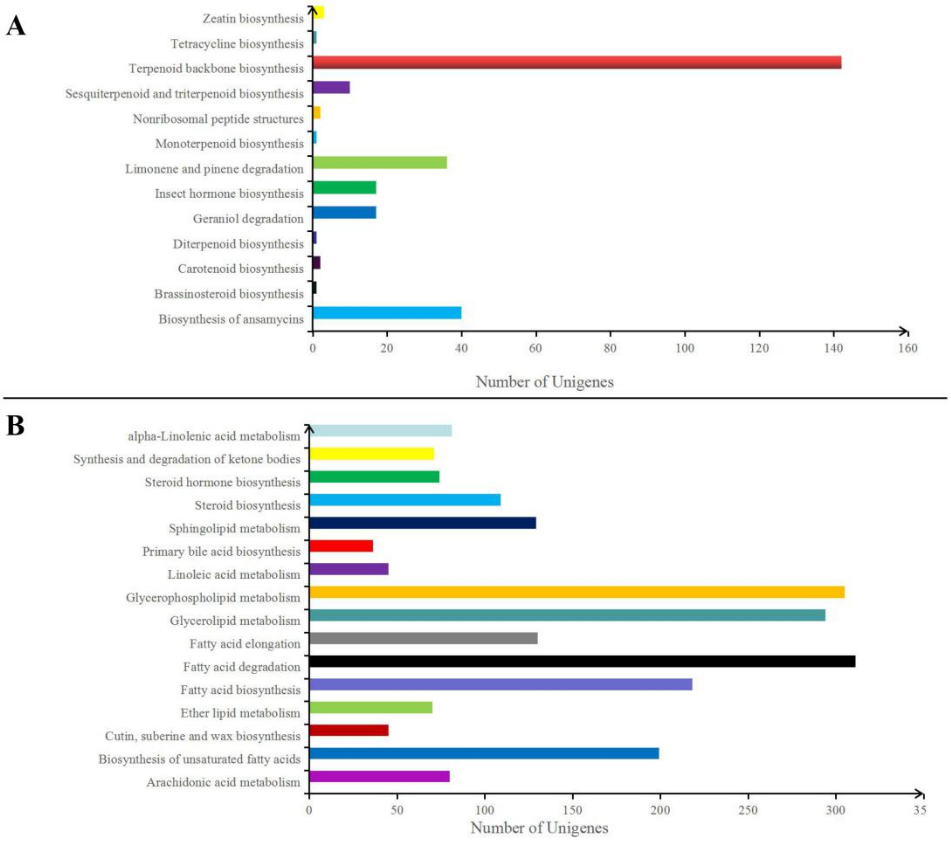

Figure S8. Metabolism of terpenoids, polyketides and lipid metabolism pathway. (A)

Metabolism of terpenoidsand polyketides pathway. **(B)** Lipid metabolism pathway. metabolism pathway is showed in Y-axis. The X-axis shows the number of genes.

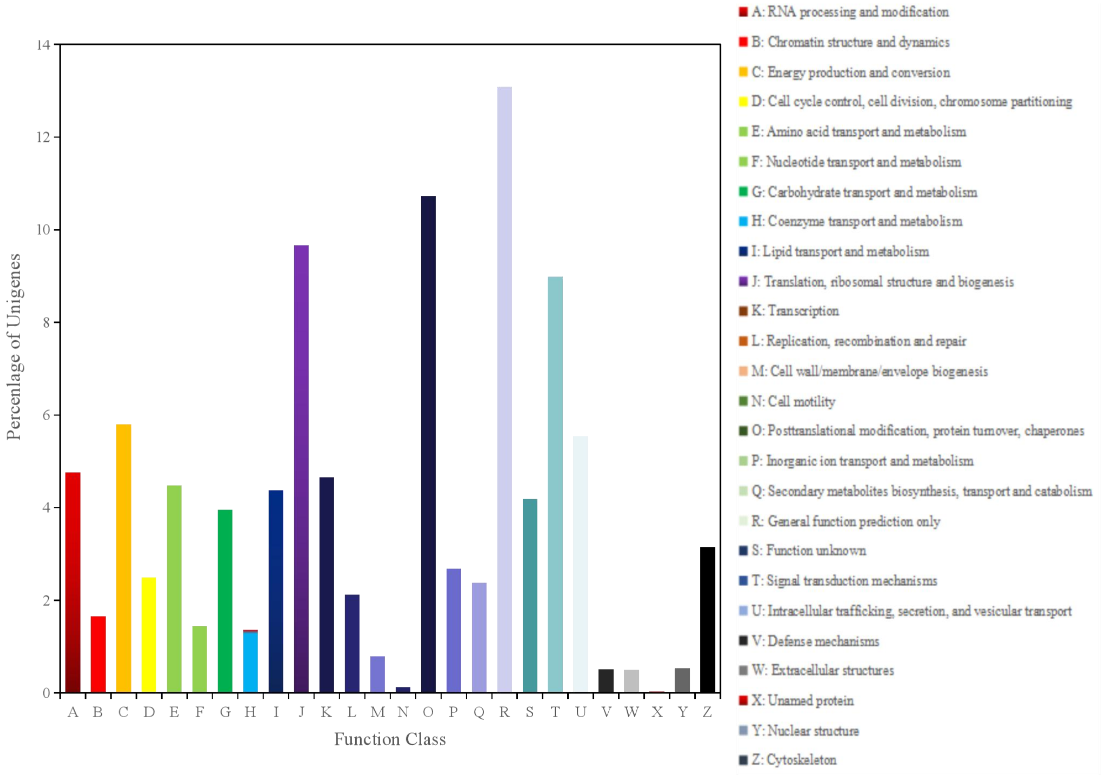

Figure S9. COG function classification of Unigenes. The horizontal coordinates are function classes of COG and the vertical coordinates are numbers of Unigenes in one class. The notaton on the right is the full name of the functions in X-axis.

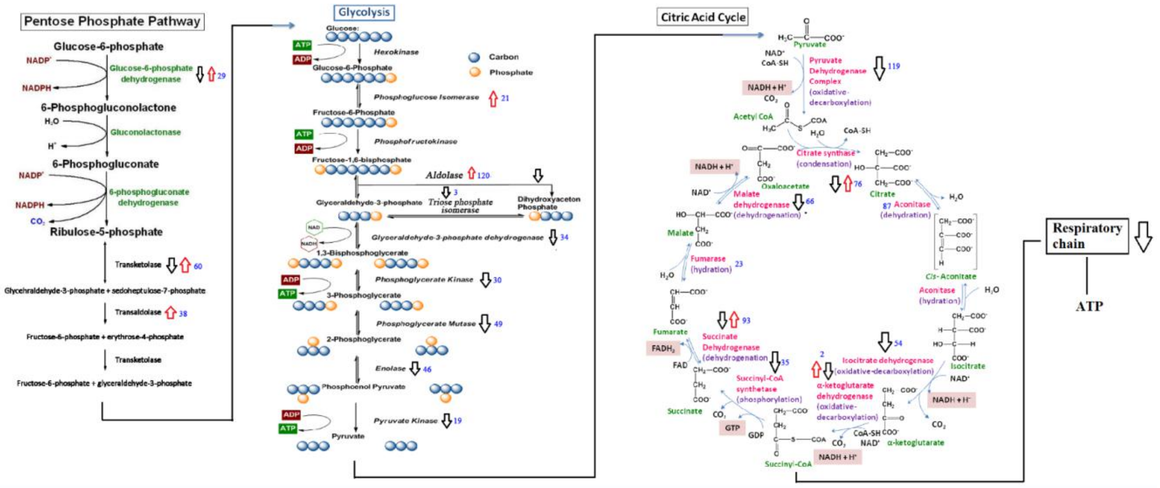

Figure S10. Central metabolic pathways. (A) Pentose phosphate patyways. **(B)** Glycolysis. **(C)** Citruate acid cycle. Blue numbers: the number of genes related to the pathways of Pentose phosphate patyways, Glycolysis and Citruate acid cycle enzymes in transcriptome data.

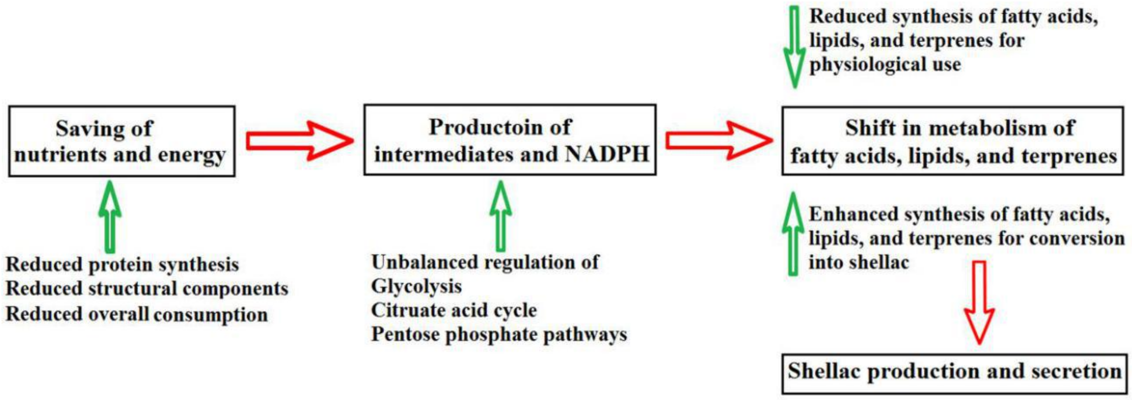

Figure S11. The lac synthesis in *K. chinensis*.

Table S1. The quantitative PCR primers of 14 candidate genes in *K. chinensis*

Gene Name	primer sequence	Tm	Gene length
FS-1	CGGAAGAGGACGAAGAAC	50.1	249
	CGCCAAGTGTAGCATAGAT	50.2	
FS-3	ATGCTTACTCCGCTATATGG	50.2	103
	AACTGGTCGTATTCGCTTAT	50.3	
FS-4	CATCGTTCTTACAAGGCTAA	48.8	142
	TATGTGGATCGGCATTCG	49.8	
FS-6	CGTCCATACCAGAGCATAA	49.5	192
	AGGATTCAGGCAGCATTC	49.7	
FS-9	TGTGACGAAGTAGCGAATA	49.1	124
	GCAACAGTTACATAGTGAGG	49.5	
FS-11	GCCGAACGAATAGCCAAT	50.7	244
	ATTACGAACAGCAGCCAAT	50.6	
FS-13	CATTCCAGAATCACGCATT	49.2	130
	CGACACAATAACCATCCTAC	49.3	
FS-14	GGTCCAATGTCATCGCTAT	50.2	167
	TGATCGGTGAGTCCATAATC	50	
FS-15	GAGCAATTCGTTGAGTTCC	50	202
	GGAGGATTACTTGAAGAATGG	49.6	
FS-16	CTCCTTCTATTCCACCAACA	50	224
	GACATTCCAGCGATACAGT	50.2	
FS-17	GTGGTGTTGCTAGTTGGTA	50.1	169
	GTGATTCCTCTGGTATCTGAT	50.1	
FS-19	CGAGCATAAGGATGACTGA	49.5	165
	AGGACAAGAAGTTACCGATT	49.6	
TBS-3	TGCTATAATGGACTGGAGAC	49.6	233
	TCACTGGTGCTGATGTTAA	49.3	
TBS-4	GCGATACACTCTATAACAGTTG	50.8	186
	GCCGTTGCTATGCTTATTG	50.5	
Actin	ATCGTGCTGAGTGAGGAA	50.6	143
	CGCTTCGCTGATTATCGTA	50.9	

Table S2. Assembly quality of *K.chinensis* transcriptome

Length range	Transcript	unigene
200-500	141 119（68.65%）	134 390（73.08%）
500-1000	35 481（17.26%）	30 241（16.44%）
1000-2000	18 510（9.0%）	13 064（7.10%）
＞2000	10 471（5.09%）	6 204（3.38%）
Total number	205 581	183 899
Total length	126 059 825	97 781 168
N50 length	910	670
Mean length	613	532

Table S3. The commonly down-regulated genes metabolic pathways

Metabolic pathways	Numbers of Unigene	Percent (%)
Protein metabolism	251	21.13
RNA metabolism	35	2.95
Structural components	52	4.38
Detoxification and defense	29	2.44
Regulators	95	8.00
Transport	44	3.70
Central and energy metabolism	73	6.14
Non-central metabolism	78	6.57
Functions unknown	42	3.54
Un-annotated genes	489	41.16

Table S4. The commonly up-regulated genes metabolic pathways

Metabolic pathways	Numbers of Unigene	Percent (%)
Protein metabolism	17	8.42
RNA metabolism	3	1.49
Structural components	10	4.95
Detoxification and defense	3	1.49
Regulators	13	6.44
Transport	20	9.90
Central and energy metabolism	12	5.94
Non-central metabolism	44	21.78
Functions unknown	8	3.96
Un-annotated genes	72	35.64

Table S5. FPKM of 28 putative genes related to Fatty acid synthesis, Terpenoid biosynthesis and UDP-driving glycosylation

Gene name	L	A1	A2	Functional categories	Pathway
FS-1	30.91	79.77	60.43	Acyl-CoA synthetase	Fatty acid synthesis
FS-2	68.93	120.59	113.01	Acyl-CoA reductase	
FS-3	9.97	72.76	49.06	Fatty acid desaturase	
FS-4	3.50	40.76	20.13		
FS-5	8.08	19.14	17.64	Fatty acid synthase	
FS-6	5.52	12.67	10.56		
FS-7	41.65	75.73	81.00	Acyl-CoA reductase	
FS-8	51.79	108.16	235.20		
FS-9	292.05	369.38	460.07	17-beta hydroxysteroid dehydrogenase	
FS-10	305.99	535.47	1117.96	ATP-citrate lyase	
FS-11	103.17	167.40	390.57	Fatty acid synthase	
FS-12	28.03	56.29	46.51		
FS-13	1347.62	1370.62	6298.32	Fatty acid desaturase	
FS-14	49.47	48.14	134.88		
FS-15	101.54	107.73	220.37	Enoyl-CoA hydratase	
FS-16	72.24	69.49	134.01	Trans-2,3-enoyl-CoA reductase	
FS-17	186.58	259.27	405.53	Medium-chain acyl-CoA dehydrogenase	
FS-18	95.65	162.36	166.55	Fatty acyl-CoA elongase	
FS-19	25.41	50.20	76.51	Acetyl-CoA carboxylase	
TBS-1	0.55	35.93	24.85	Polyprenyl synthetase	Terpenoid biosynthesis
TBS-2	0.27	20.47	15.29		
TBS-3	35.05	93.83	32.48	Decaprenyl-diphosphate synthase	
TBS-4	76.62	130.01	335.93	Hydroxymethylglutaryl-CoA synthase	
UDP-1	76.52	120.96	157.81	Oligosaccharyltransferase	UDP-driving glycosylation
UDP-2	14.69	32.42	96.38	UDP-glucuronosyl/-glucosyl transferase	
UDP-3	20.18	83.74	102.36		
UDP-4	118.44	190.31	360.64		
UDP-5	10.96	25.15	36.90		
